# Heterogeneity of resistant mechanisms in an *EGFR*-TKI relapsed patient with *EGFR* amplification and response to nimotuzumab: A case report

**DOI:** 10.3389/fonc.2022.937282

**Published:** 2022-08-11

**Authors:** Yan Li, Ziyi Xu, Tongji Xie, Puyuan Xing, Jianming Ying, Junling Li

**Affiliations:** ^1^ Department of Pathology, National Cancer Center/National Clinical Research Center for Cancer/Cancer Hospital, Chinese Academy of Medical Sciences and Peking Union Medical College, Beijing, China; ^2^ Department of Medical Oncology, National Cancer Center/National Clinical Research Center for Cancer/Cancer Hospital, Chinese Academy of Medical Sciences and Peking Union Medical College, Beijing, China

**Keywords:** case report, resistance, heterogeneity, EGFR amplification, nimotuzumab

## Abstract

*EGFR* mutations are the most important drivers of gene alterations in lung adenocarcinomas and are sensitive to *EGFR*-TKIs. However, resistance to *EGFR*-TKIs is inevitable in the majority of *EGFR*-mutated lung cancer patients. Numerous resistant mechanisms have been revealed to date, and more are still under investigation. Owing to the selective pressure, intratumoral heterogeneity may exist after resistance, especially in patients after multiple lines of treatment. For those patients, it is important to choose therapies focused on the trunk/major clone of the tumor in order to achieve optimal clinical benefit. Here, we will report an *EGFR*-mutated lung adenocarcinoma patient with heterogeneity of resistant mechanisms including *EGFR* amplification, large fragment deletion of *RB1*, and histological transformations after targeted treatments. In our case, *EGFR* amplification seemed to be the major clone of the resistant mechanism according to the next-generation sequencing (NGS) results of both liquid biopsy monitoring and tissue biopsies. In consideration of the high *EGFR* amplification level, the patient was administered by combination treatment with *EGFR*-TKI plus nimotuzumab, an anti-*EGFR* monoclonal antibody (mAb), and achieved a certain degree of clinical benefit. Our case sheds light on the treatment of *EGFR*-mutant patients with *EGFR* amplification and indicates that a combination of *EGFR*-TKI with anti-*EGFR* mAb might be one of the possible treatment options based on genetic tests. Moreover, the decision on therapeutic approaches should focus on the major clone of the tumor and should make timely adjustments according to the dynamic changes of genetic characteristics during treatment.

## Introduction

*EGFR* mutations are the most important driver of gene alterations in lung adenocarcinoma (LUAD) patients, especially in Asian non-smoking women (1). *EGFR*-TKIs are normally highly effective in patients with *EGFR* mutations. However, acquired resistance would eventually occur in the majority of patients. Among multiple mechanisms leading to *EGFR*-TKI resistance, the T790M mutation is most commonly found in subsequent first- and second-generation *EGFR*-TKIs (2, 3). Other resistant mechanisms, such as bypass activation and histological transformations, have also been reported (2, 4–6). Moreover, individual reports have also indicated that *EGFR* amplification contributes to acquired resistance to *EGFR*-TKIs (7–9). According to the AURA study, *EGFR* amplification occurred in 5.3% (1/19) of patients who developed resistance to first-line osimertinib (10). *EGFR* amplification was found in 42.9% (3/7) of T790M-positive tissues after resistance to third-generation tyrosine kinase inhibitor (TKI) (11).

Owing to the selective pressure, intratumoral heterogeneity may exist after resistance, especially in patients after multiple lines of treatment. Overcoming tumor heterogeneity is a major challenge for cancer treatment. Several studies have demonstrated that higher levels of heterogeneity in lung cancer predict inferior responses to anticancer therapies, including targeted therapy and immunotherapy (12, 13). For patients with intratumoral heterogeneity, it is important to choose therapies focused on the trunk/major clone in the tumor to achieve optimal clinical benefit. Herein, we report a LUAD patient with heterogeneity of resistant mechanisms to *EGFR*-TKIs, mainly based on *EGFR* amplification, who was successfully treated with a combination treatment containing nimotuzumab and *EGFR*-TKIs subsequently. We will present the following case in accordance with the CARE reporting checklist.

## Case description

**Figure 1:**
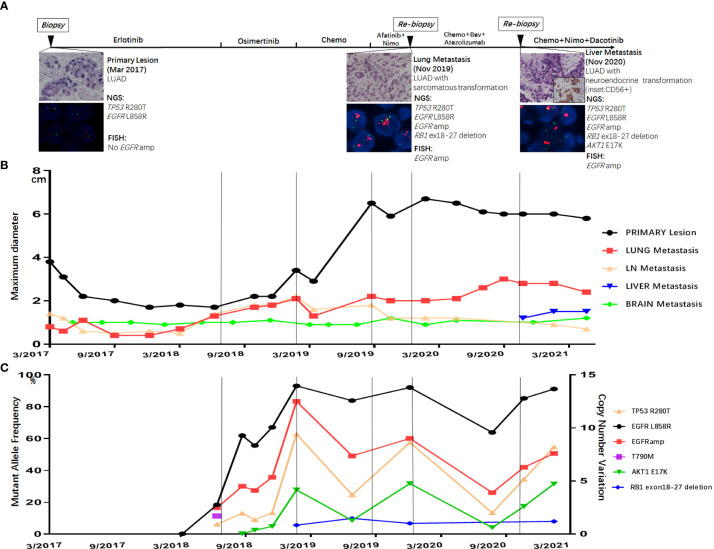
**(A)** Treatment timeline of the patient, histological morphology, and genetic results of three tissue biopsies (primary lesion in March 2017, lung metastasis in November 2019, and liver metastasis in November 2020). Dynamic monitoring of radiological results **(B)** and circulating tumor DNA using next-generation sequencing (NGS) **(C)**.

A 53-year-old Chinese man was found to have an occupying lesion in the upper lobe of the right lung during a routine checkup in March 2017. Positron emission tomography–computed tomography (PET-CT) showed multiple metastatic nodules in the hilum of the lung, lymph nodes, bone, and brain. The patient underwent tissue biopsy through a bronchoscope and was diagnosed with stage IV LUAD (**Figure 1A**). Next-generation sequencing (NGS) was performed using the tissue for driver gene alteration testing and revealed mutations of *EGFR* L858R and *TP53* R280T. Physical examination in this patient suggested no significant abnormalities except that the Karnofsky performance status (KPS) was 70. Laboratory findings were within the normal range, except for the carcinoembryonic antigen (CEA) level of 10.87 ng/ml (normal range, 0 to 5 ng/ml) in the serum. Other exams showed no positive signs at diagnosis.

The patients started with erlotinib 150 mg once daily as first-line therapy. After 16 months of erlotinib treatment, CT scans showed progressed disease (PD) due to the enlargement of lung metastatic nodules (**Figure 1B**). Liquid biopsy using plasma detected T790M along with L858R mutation, as well as *EGFR* amplification (**Figure 1C**). The patient started osimertinib treatment at 80 mg once daily as second-line therapy, and liquid biopsy monitoring only detected L858R and *EGFR* amplification 2 months after osimertinib, with no T790M retained. Osimertinib treatment lasted for 7 months before the resistance occurred (**Figure 1B**). Liquid biopsy after osimertinib resistance showed L858R mutation with high *EGFR* amplification. Moreover, *AKT1* E17K and a large fragment deletion of *RB1* (exon18–27) were also detected (**Figure 1C**). The patient then received pemetrexed plus platinum for six cycles. CT scans showed rapid growth of the primary lesion in September 2019 (**Figure 1B**). Taking the high level of *EGFR* amplification into consideration, the patient was administered by combination treatment with afatinib plus nimotuzumab and had stable disease (SD) with a slight shrinking of all the tumor sites including the primary and metastatic lesions. The combination treatment of afatinib and nimotuzumab lasted for 3.7 months, and then the patient underwent a re-biopsy in November 2019. Tissue biopsy guided by CT of metastatic lung lesion showed LUAD with sarcomatoid differentiation (**Figure 1A**). Then the patient asked for chemotherapy plus immunotherapy (bevacizumab + Abraxane + atezolizumab), and the therapy lasted for 10.4 months. In November 2020, a new liver metastatic lesion was found and was confirmed to be adenocarcinoma with neuroendocrine differentiation *via* tissue biopsy (**Figure 1A**). The NGS test showed different results in the lung and liver metastatic lesions: only the liver lesion was detected with *AKT1* E17K, while both harboring L858R, *TP53* R280T, *EGFR* amplification, and a large fragment deletion of *RB1* (exon18–27). Since then, the patient received a combination treatment of irinotecan, nimotuzumab, and dacomitinib with optimal efficacy of SD. The combination therapy has caused grade 3 hepatic disorders, represented as increased alanine aminotransferase (ALT), aspartate aminotransferase (ALP), and aspartate aminotransferase (AST) compared to baseline examination before the treatment, based on the Common Terminology Criteria for Adverse Events (CTCAE; version 5.0).

**Figure 2:**
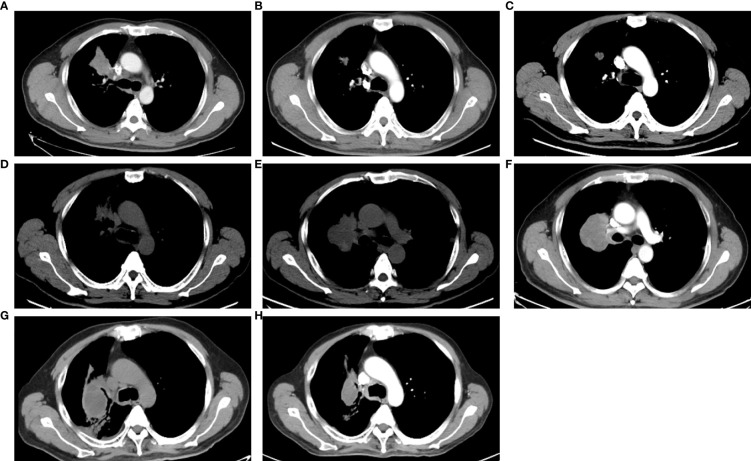
The images of CT scans with different treatments. **(A)** CT scan of basic examination before first-line erlotinib. **(B)** CT scan during the first-line erlotinib treatment. **(C)** CT scan at the progression of first-line erlotinib. **(D)** CT scan at progression of second-line osimertinib. **(E)** CT scan at the progression of chemotherapy. **(F)** CT scan at progression of afatinib plus nimotuzumab. **(G)** CT scan at the progression of immunotherapy. **(H)** CT scan during combination therapy of irinotecan, nimotuzumab, and dacomitinib.

*EGFR* amplification was validated by fluorescence *in situ* hybridization (FISH) *via* tissue biopsies, including pre-treatment biopsies from primary tumors and post-treatment re-biopsies from lung and liver metastasis lesions. FISH signal accounts (copy number) were recorded for a total of 50 nuclei, and the tumor was considered *EGFR* amplification when *EGFR*/*CEP7* ratio was greater than or equal to 2 in 15% of recorded cells (14). **Figure 1A** shows that FISH revealed *EGFR* amplification in the metastatic lesions of both lung and liver, while no *EGFR* amplification was observed in the primary lesion. Dynamic monitoring of radiological results and liquid biopsy NGS results are shown in **Figures 1B, C**. The study was approved by the institutional review board of the Cancer Hospital, Chinese Academy of Cancer Science (CAMS). Written informed consent was signed by the patient. The images of chest CT scans at different timepoints are shown in **Figure 2**.

The combination treatment of irinotecan, nimotuzumab, and dacomitinib remained for 8.6 months with continued disease control before the patient died in July 2021 due to tumor progression and complications. The PFS of different treatments is listed in **Table S1**.

## Discussion

**Figure 3:**
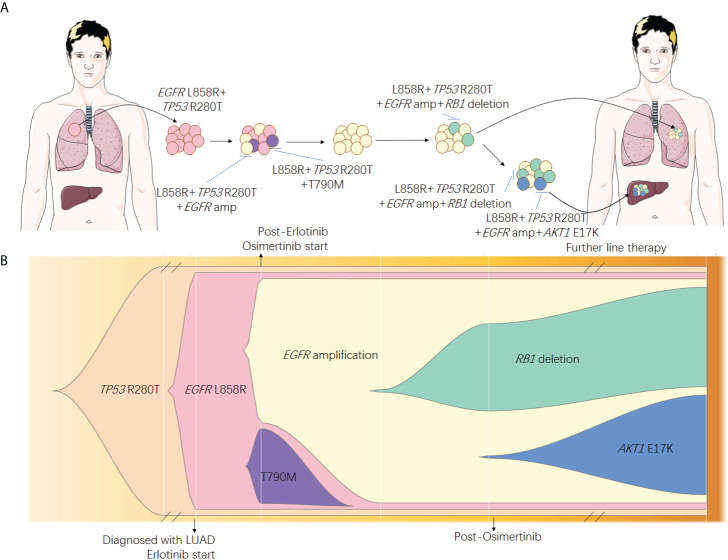
Speculated spatial heterogeneity **(A)** and evolutionary spectrum **(B)** of the patient.

Resistance to *EGFR*-TKIs is inevitable, and numerous mechanisms have been discovered to date. Previously studies have described heterogeneity of resistant mechanisms after *EGFR*-TKI treatment. Roper et al. reported that 73% of osimertinib relapsed patients harbored at least two co-existing resistant mechanisms (15). Chabon et al. reported that intra-patient heterogeneity was observed in 46% of patients after first-line *EGFR*-TKI and 33% of patients after osimertinib treatment, according to circulating tumor DNA analysis (12). Our case emphasized the tumor spatial and temporal heterogeneity (**Figure 3**) by adequate investigation of tissue biopsies and liquid biopsy monitoring. Although the resistant mechanisms showed heterogeneity in our patients, *EGFR* amplification seemed to be the major clone of the resistant mechanism. Since *EGFR* amplification was observed early in liquid biopsy monitoring and all tissue biopsies after resistance harbored *EGFR* amplification, we speculated that other resistant mechanisms, such as large fragment deletion of *RB1* and histological transformation, were all divergent propagation of subclones from *EGFR* amplification. To achieve the optimal clinical benefit, it is important to choose therapies focused on *EGFR* amplification for this patient. Combination approaches that target heterogeneous tumor clones have been proved to be successful in pre-clinical studies (16). Nimotuzumab, an anti-*EGFR* monoclonal antibody (mAb), was used to treat *EGFR* overexpression cancers including glioma (17), squamous cell carcinomas of the head and neck (18), non-small cell lung cancer (NSCLC) (19), and other tumors of epithelial origin (20). It was also reported to have superior antitumor activity when combined with *EGFR*-TKI such as afatinib (21) in *EGFR*-mutant lung cancer. In our case, combination treatment of *EGFR*-TKI (afatinib or dacomitinib) plus nimotuzumab showed efficacy to some extent and significantly inhibited the rapid growth of both primary and metastasis lesions (**Figure 1B**). Other *EGFR* mAbs have been also evaluated in lung cancer for a long time, and *EGFR* amplification may be a predictive factor of *EGFR* mAb such as cetuximab and necitumumab plus chemotherapy (22, 23). In gastrointestinal cancers such as colorectal cancer, panitumumab, another *EGFR* mAb, has also been proved to be effective for patients with *EGFR* amplification (24, 25). However, evidence of the combination treatment of EGFR mAb plus EGFR-TKI for patients with both *EGFR* mutation and amplification has been scarce.

It is noticed that patients underwent chemotherapy plus immunotherapy (bevacizumab + Abraxane + atezolizumab) for 10.4 months subsequent to standard treatment and received stable disease. The relationship between immunotherapy and driver mutations has long been a research hotspot. A previous meta-analysis study showed that immunotherapy could not enhance overall survival (OS) or progression-free survival (PFS) in *EGFR*-mutant patients (26). At the same time, IMpower 150 reported a prolonged OS in *EGFR*-mutated patients with atezolizumab plus chemotherapy and bevacizumab (27). In our study, the *EGFR*-mutant patient did benefit from immunotherapy after multiple lines of treatments. Thus, further studies are needed to effectively evaluate the efficacy of immunotherapy for NSCLC individuals with *EGFR* mutations.

Our case sheds light on the treatment of *EGFR*-mutant patients with *EGFR* amplification and indicates that a combination of *EGFR*-TKI with anti-*EGFR* mAb might be one of the possible treatment options. Moreover, as there are more and more treatment options for LUAD, patients could survive with tumors for a longer time. It brings up another problem that intratumoral heterogeneity might be more complicated after multi-line treatments. Thus, importance should be attached to therapeutic approaches to the major clone of the tumor, and these approaches should be multidimensional and dynamic with timely adjustments according to the patients’ genetic characteristics.

## Data availability statement

The raw data supporting the conclusions of this article will be made available by the authors, without undue reservation.

## Ethics statement

The studies involving human participants were reviewed and approved by institutional review board of the Cancer Hospital, CAMS. The patients/participants provided their written informed consent to participate in this study.

## Author contributions

JL was the patient’s physician, proposed the conception, collected the data for case presentation. YL conducted the literature search, performed the NGS tests for tissues and liquid biospies, and prepared the first draft of the manuscript. JY contributed to the conceptualization and contributed with final manuscript drafting. ZX conducted the follow-up and the revision of manuscript. TX was responsible for data analysis and figure drawing. PX contributed with final manuscript drafting. All authors contributed to the article and approved the submitted version.

## Funding

This work was supported by a grant from the National Natural Science Foundation of China (NSFC No. 81802294).

## Acknowledgments

We also would like to thank the patient and his family for consenting to the present publication.

## Conflict of interest

The authors declare that the research was conducted in the absence of any commercial or financial relationships that could be construed as a potential conflict of interest.

## Publisher’s note

All claims expressed in this article are solely those of the authors and do not necessarily represent those of their affiliated organizations, or those of the publisher, the editors and the reviewers. Any product that may be evaluated in this article, or claim that may be made by its manufacturer, is not guaranteed or endorsed by the publisher.
